# Stoichiometry and kinetics of single and mixed substrate uptake in *Aspergillus niger*

**DOI:** 10.1007/s00449-017-1854-3

**Published:** 2017-10-19

**Authors:** Francisca Lameiras, Cor Ras, Angela ten Pierick, Joseph J. Heijnen, Walter M. van Gulik

**Affiliations:** Cell Systems Engineering section, Department of Biotechnology, Delft University of Technology, 2629 HZ Delft, The Netherlands

**Keywords:** *Aspergillus niger*, Plant waste streams, Substrate uptake, Kinetics, Stoichiometry

## Abstract

In its natural environment, the filamentous fungus *Aspergillus niger* grows on decaying fruits and plant material, thereby enzymatically degrading the lignocellulosic constituents (lignin, cellulose, hemicellulose, and pectin) into a mixture of mono- and oligosaccharides. To investigate the kinetics and stoichiometry of growth of this fungus on lignocellulosic sugars, we carried out batch cultivations on six representative monosaccharides (glucose, xylose, mannose, rhamnose, arabinose, and galacturonic acid) and a mixture of these. Growth on these substrates was characterized in terms of biomass yields, oxygen/biomass ratios, and specific conversion rates. Interestingly, in combination, some of the carbon sources were consumed simultaneously and some sequentially. With a previously developed protocol, a sequential chemostat cultivation experiment was performed on a feed mixture of the six substrates. We found that the uptake of glucose, xylose, and mannose could be described with a Michaelis–Menten-type kinetics; however, these carbon sources seem to be competing for the same transport systems, while the uptake of arabinose, galacturonic acid, and rhamnose appeared to be repressed by the presence of other substrates.

**Electronic supplementary material:**

The online version of this article (doi:10.1007/s00449-017-1854-3) contains supplementary material, which is available to authorized users.

## Introduction

In white biotechnology, the feedstock represents the highest cost factor in the production of bulk chemicals. Therefore, there is an increasing interest in using cheaper biomass streams as feedstock for industrial biotechnology processes, the so-called second-generation feedstocks.

The second-generation feedstocks have the advantage that they do not compete with food supplies, but have the disadvantage that they are much more complex than first-generation ones. The second-generation feedstocks consist of mixtures of different fermentable carbon sources from plant biomass of agricultural crops waste, which are currently insufficiently used. Of the global 200 × 10^9^ tons per year of plant biomass produced, over 90% is lignocellulose. About 8–20 × 10^9^ tons of this biomass is potentially accessible, but remains unexploited [1].

In [2], the performance of six industrially relevant microorganisms was tested by submitting them to growth conditions that they encounter in a second-generation feedstock-based production process (mixture of substrates, inhibitors, extreme pH, etc.). The generated data were used to rank the organisms by relative performance, and *Aspergillus niger* scored the highest, leading to a stronger motivation for the use of this microorganism in the second-generation feedstocks [2].

*A. niger* is known to grow on hexose as well as pentose substrates, and can also hydrolyse plant material due to high levels of excreted enzymes having the capacity to degrade the plant cell wall polysaccharides [3].

Lignocellulose contains three structural polysaccharides: cellulose (40–50%), hemicellulose (25–35%), and lignin (15–20%) [4]. Besides glucose, sugar monomers in hemicellulose can include xylose, galactose, mannose, and arabinose; pectins on the other hand are rich in galacturonic acid and rhamnose.

The shift of industrial biotechnology from highly refined sugar syrups (first-generation feedstocks) to more sustainable and cheaper carbon and energy sources requires a shift from single substrate to mixed substrate use [5].

To obtain a better understanding of the uptake of individual substrates in a complex mixture as well as the stoichiometry and kinetics of growth and product formation under those conditions, careful kinetic studies are required. In general, the uptake and metabolism of different carbon sources in heterotrophic microorganisms are controlled through carbon catabolite repression (CCR). In many cases, glucose is the preferred substrate for growth, and thus, the presence of glucose will repress the uptake and metabolism of other available carbon sources. Glucose repression was first studied in the bacterium *Escherichia coli* [6] and later on also in yeasts and filamentous fungi [7].

In this work, we studied the capacity of *A. niger* for uptake of six-selected monosaccharides present in lignocellulosic biomass, that is, three C6 sugars (glucose, mannose, and rhamnose) two C5 sugars (xylose and arabinose), and a sugar acid (galacturonic acid). We investigated the stoichiometry and kinetics of substrate uptake and aerobic growth on these different carbon sources, under substrate limited (sequential chemostat cultures) as well as substrate excess conditions (shake flask and batch cultivations), using single and mixed substrate feedstocks. With the application of *A. niger* for the production of organic acids in mind, we used a strain (*A. niger* NW185) not capable of producing gluconic and oxalic acid and carried out all cultivations at pH 2.5.

## Materials and methods

### Strain and inoculum

The strain used was *A. niger* NW185 (*cspA1* short conidiospores, *fwA1* fawn coloured spores, *goxC17* glucose oxidase negative, and *prtF28* oxalate non-producing). Unless stated otherwise, the hyphal inocula for batch and continuous cultivations were obtained from shake flask cultures. Spores for pre-inoculum were grown on agar plates and harvested, as described in [8]. A concentration of 1 × 10^6^ spores/mL was pre-inoculated in a 250 mL shake flask containing 100 mL of PM medium and 100 mM of sorbitol [9] at pH 4.5. Sorbitol was used as a pre-substrate for the pre-cultivation as it does not induce or repress any catabolic system [10].

The shake flask was incubated overnight at 30 °C in an orbital shaker (CERTOMAT BS-1, Sartorius group) at 250 rpm. The resulting mycelia suspension was separated by centrifugation (*Heraeus Biofuge Stratos, Thermo Scientific*, USA) for 2 min at 10000G, 4 °C and washed two times with 50 mL PM medium without carbon source at room temperature and subsequently used to inoculate the bioreactor (one shake flask per bioreactor).

### Shake flask cultivation

*A. niger* was inoculated in 250 mL shake flasks, containing 100 mL minimal medium [0.5 g/L (NH_4_)_2_SO_4_, 0.3 g/L KH_2_PO_4_, 3 g/L NH_4_H_2_PO_4_ and 0.5 g/L MgSO_4_·7H_2_O], at initial pH 2.5. The pH 2.5 for growth in continuous cultivation has high advantages in terms of morphology and broth homogeneity [8], but is also highly relevant because we are interested in organic acid production.

In each shake flask, the medium was supplemented with one single carbon source, such that the added amount of carbon was the same (total 228 mCmol/L), namely, 38 mM of d-glucose, d-galacturonic acid, l-rhamnose and d-mannose, and 45.6 mM of d-xylose and l-arabinose. Furthermore, 1 mL/L of trace elements solution was added, containing 10 g/L EDTA, 4.4 g/L ZnSO_4_·7H_2_O, 1.0 g/L MnCl_2_·4H_2_O, 0.32 g/L CoCl_2_·6H_2_O, 0.32 g/L CuSO_4_·5H_2_O, 0.22 g/L (NH_4_)_6_Mo_7_O_24_·4H_2_O, 1.47 g/L CaCl_2_·2H_2_O, and 1.0 g/L FeSO_4_·7H_2_O [11].

Medium was sterilized at 121 °C for 20 min and the carbon-source solutions were sterilized separately at 110 °C. The trace elements were added sterile to the culture media by filtration through a 0.2 µm cartridge filter (Whatman FP 30/0.2 CA-S).

One set of shake flasks was inoculated with spores and the second set with washed mycelia from one shake flask previously grown in PM medium as described above.

Cultures were incubated for 50 h in an orbital shaker at 30 °C and 250 rpm (CERTOMAT BS-1, Sartorius group).

### Single carbon-source batch cultivation

Batch cultivations were operated in a 2 L bioreactor with 1 L working volume (Applikon, Schiedam, The Netherlands), on minimal medium (see previous section) supplemented with either 38 mM d-glucose, 38 mM d-galacturonic acid, 38 mM l-rhamnose, 38 mM d-mannose, 45.6 mM of d-xylose or 45.6 mM l-arabinose.

The bioreactor was equipped with pH, temperature, and dissolved oxygen probes. Throughout the cultivation, the pH was kept at 2.5 (± 0.1) by controlled addition of 1M NaOH and the temperature was controlled at 30 °C (± 0.1).

Sterile air was supplied via a mass flow controller (Brooks 58505 calibration at 0 °C and 1 bar) at 0.22 vvm through a bottom sparger, and an overpressure of 0.3 bar was applied.

The exhaust gas of the fermentor was passed through a condenser (4 °C) and a Nafion dryer (Permapure, Toms River, USA) before entering an NGA 2000 offgas analyser (Rosemount Analytical, Anaheim, USA) for measurement of the oxygen and carbon dioxide contents. Data acquisition was performed with MFCS/win 3.0 software.

### Mixture of carbon-source batch cultivation

Fermentation conditions and control were executed as described for the single substrate batch fermentations (previous section), with the difference that the minimal medium was supplemented with equimolar concentrations of carbon of six different carbon sources (6.3 mM d-glucose, 6.3 mM d-galacturonic acid, 6.3 mM l-rhamnose, 6.3 mM d-mannose, 7.6 mM of d-xylose, and 7.6 mM l-arabinose), to a total of 228 mCmol/L.

### Sequential chemostat cultures

In contrast to the batch fermentations (previous two sections), the sequential chemostat culture experiment was performed in a 7 L bioreactor with a working volume of 4.5 L Fermentor set up and operating conditions were the same, as described in [8], and temperature and pH were the same as the batch experiments.

The reactor was inoculated with pre-grown and washed mycelia into minimal media supplemented with 12.6 mM d-glucose, 12.6 mM d-galacturonic acid, 12.6 mM l-rhamnose, 12.6 mM d-mannose, 15.2 mM of d-xylose, and 15.2 mM l-arabinose. After the batch phase was finished, the feed was initiated with a starting dilution rate of 0.04 h^−1^. Subsequently, the dilution rate was increased to 0.21 h^−1^ in 11 steps (0.04, 0.06, 0.08, 0.09, 0.11, 0.13, 0.14, 0.16, 0.17, 0.19, and 0.21 h^−1^).

### Sampling and analytical procedures

During batch cultivations (both single and mixture of substrates), at least six samples (in triplicates) at different timepoints were taken for quantification of cell dry weight, biomass elemental composition, extracellular metabolites, and total organic carbon (TOC), during the exponential phase and at the end of the batch. During the sequential chemostat cultures experiment, triplicate samples were taken after two residence times, at each dilution rate.

Biomass dry weight was determined by filtration of 5 mL of broth over glass fiber filters (47 mm, type A/E, Pall, USA), subsequent washing with 10 mL of water and drying at 70 °C until constant weight. All samples were analysed in quadruplicate.

Biomass elemental composition was determined by combustion and subsequent gas analysis (carbon dioxide, water vapour, and nitrogen mass fractions), gas chromatography (oxygen), and ICP-MS (phosphorus and sulphur) (Energy Research Centre of the Netherlands).

Filtrate samples were obtained by quickly (within 3 s) withdrawing 5 mL of broth, via the overpressure on the fermentor, into a syringe containing cooled steel beads (− 20 °C) to bring the sample temperature down to 0 °C [12]. The broth was then immediately pressed through a 0.45 µm cartridge filter (Millex-HV durapore PVDF membrane) into a sampling vial, which was directly frozen in liquid nitrogen.

The total organic carbon content of filtrate samples was quantified with a total organic carbon analyser (type TOC-L, Shimadzu, Kyoto, Japan). With this method, both total carbon (TC) and inorganic carbon (TIC) were measured. The latter representing the content of dissolved carbon dioxide and carbonic acid salts. Subtracting the inorganic carbon from the total carbon, yielded the TOC in filtrate.

The residual concentration of the different carbon sources was measured by different methods.

For the batch cultivation on single substrates, analyses were implemented by an HPLC method, using an H^+^ exchange column at 60 °C (Bio-Rad HPX-87H 300 × 7.8 mm), employing phosphoric acid 1.5 mmol/L in Milli-Q water at 70 °C as mobile phase with a refractive index detection (Waters 2414; sens = 1024; temp = 30 °C) and UV detection (Waters 2489; 210 nm).

For the mixture of carbon sources, both GC-IDMS/MS and HPLC methods were used. The concentrations of the sugars measured by isotope dilution mass spectrometry (GC-IDMS/MS) were according to the protocols of [13] with the following changes: temperature gradient was slower to have a better separation for xylose and arabinose and the temperature was increased with 5 °C/min instead of 10 °C/min. Glucose^13^ xylose^13^ mannose^13^ and arabinose^13^ were added as internal standards (IS) for the same ^12^C compounds, while for rhamnose and galacturonic acid, glucuronic acid and fumarate were used as IS respectively. The HPLC method used for the same measurements was performed in an ion-exchange column in lead (Pb) form column at 85 °C (Bio-Rad HPX-87P300 × 7.8 mm), employing milliQ water at 70 °C as mobile phase with a refractive index detection (Waters 2414; sens = 1024; temp = 30 °C) and UV detection (Waters 2489; 210 nm).

The samples from the sequential chemostat cultivation experiment were analysed with a Dionex ICS-5000 HPIC system with AS-AP sampler, SP pump, Carbopac PA-20 3 × 150 mm column and Aminotrap 3 × 30 mm precolumn employing 2 mM NaOH isocratic (for 17.5 min followed by a cleaning step of 5 min with 200 mM and equilibration for 15 min), 20 mM NaOH isocratic (15 min followed by 5 min cleaning with 200 mM NaOH and equilibration for 15 min) or gradient: 15 min 10 mM NaOH followed by a linear increase to 200 mM in 15 min, then a linear gradient in 20 min with 0.5 M sodium acetate in 200 mM NaOH from 4 to 40% v/v, followed by a cleaning step of 5 min 200 mM NaOH and equilibration for 15 min (for acidic sugars). All eluents with a flow of 0.5 mL/min at 30 °C. The detection was by electrochemical pulse at 15 °C.

### Balances, biomass specific rates, and data reconciliation during single carbon-source batch cultivation

Using the gas-phase balances (O_2_ and CO_2_) and liquid phase balances (biomass and substrate), the batch cumulative amounts of consumed O_2_ and substrate and produced CO_2_ and biomass were calculated.

Subsequently, because the carbon and degree of reduction balances were close to 100%, the element reconciled cumulative amounts were calculated as function of time, as described in [14].

These reconciled amounts were used to calculate the maximum biomass specific conversion rates (*μ* ^max^, *q*_S_^max^_,_ ${q_{{\text{C}}{{\text{O}}_{\text{2}}}}}^{{{\text{max}}}}$_,_ ${q_{{{\text{O}}_{\text{2}}}}}^{{{\text{max}}}}$, i.e., the biomass specific conversion rates of biomass, substrate, carbon dioxide, and oxygen, respectively) and the growth stoichiometric values.

The biomass elemental composition was analysed for each substrate and taken into account for the data reconciliation.

### Balances, biomass specific rates, and data reconciliation during sequential chemostat cultivation

Using the steady-state mole balances, the rates of substrate (*R*_S_), biomass (*R*_X_), carbon dioxide (${q_{{\text{C}}{{\text{O}}_{\text{2}}}}}$), oxygen (${q_{{{\text{O}}_{\text{2}}}}}$), and by-products in the culture filtrate (*R*_TOC_) were calculated from the primary measurements of concentrations in gas and liquid phases, as well as gas and liquid flow rates. From these rates, carbon and degree of reduction recoveries Eqs. (1), (2) were calculated:

1 $${\text{Carbon recovery}}~\left( \% \right)=~\frac{{{R_{\text{x}}}+~{R_{{\text{C}}{{\text{O}}_2}}}+~{R_{{\text{toc}}}}}}{{\Sigma ~{R_{\text{s}}}}}~ \times 100~$$

2 $${\text{Degree of reduction}}\left( {\text{\%}} \right)={\text{}}\frac{{{\gamma _x}~~~{R_{x~~~}}+~~~{\gamma _{{\text{toc}}}}~~~{R_{{\text{toc}}}}~~+{\gamma _{{{\text{O}}_2}}}~~~{R_{{{\text{O}}_2}}}}}{{\Sigma ~{\gamma _{\text{s}}}~{R_{\text{s}}}}} \times 100$$

where Σ*R*_S_ is the weighted sum of all consumed carbon sources in Cmol/L, and *R*_TOC_ is the residual total organic carbon resulted from the subtraction of the concentration of the residual substrates to the measured TOC. After checking absence of relevant carbon and degree of reduction gaps, data reconciliation was applied to obtain the best estimates of the measurements, within their error margins, using the elemental and charge conservation relations as constraints [15].

### Substrate uptake kinetics

Microbial growth studies report several different equations to define substrate uptake kinetics. The most simple and widely used equations follow the hyperbolic Michaelis–Menten model of enzyme kinetics Eq. (3):

3 $${q_{\text{S}}}={q_{{\text{Smax}}}} \times \frac{{{C_{\text{S}}}}}{{{K_{\text{S}}}+{C_{\text{S}}}}}$$

In a first approach of this study, the data obtained from the sequential chemostat cultivations were fitted to a Michaelis–Menten equation. When *C*_S_ = *K*_S_, the value of q_S_ corresponds to 50% of the *q*_S_^max^.

Depending on the outcome of the fitting of Eq. (3), two situations were considered: competition for the same transport system or catabolite repression from other substrates.

To model the uptake of competing sugars, we assumed that these sugars are taken up by the same import systems and that their uptake could be described by competition, according to [16]—Eq. (4):

4 $${v_{1=\frac{{{v_{{\text{max1}}}}{S_1}}}{{{K_{m1}}\left( {1+\mathop \sum \nolimits_{{i=2}}^{n} \frac{{{S_i}}}{{{K_{mi}}}}} \right)+{S_1}}}}}$$

where *S*_1_ competes with *n* − 1 substrates *S*_2_,…,*n* for the binding site of the enzyme. The variables *K*_mi_ describe the Michaelis–Menten affinity constant for each subtract *S*_i_.

Catabolite repression of the uptake/metabolism of different carbon sources can be described by a so-called switch function, based on the Hill kinetics (Eq. 5), whereby the expression of an enzyme (e.g., transport protein or rate controlling enzyme of a catabolic pathway) is repressed by the presence of another carbon source:

5 $${v_{{\text{max}}}}\left( R \right)={v_{{\text{max}}}}\left( U \right)\left( {\frac{1}{{1+{{\left( {\frac{R}{{{K_{\text{R}}}}}} \right)}^n}}}} \right)$$

Herein, *v*_max_(*R*) and *v*_max_(*U*) are, respectively, the repressed and unrepressed enzyme rates, *R* is the repressor concentration, and *K*_R_ is the repression constant.

## Results and discussion

### Shake flask cultivations on single carbon sources

**Fig. 1:**
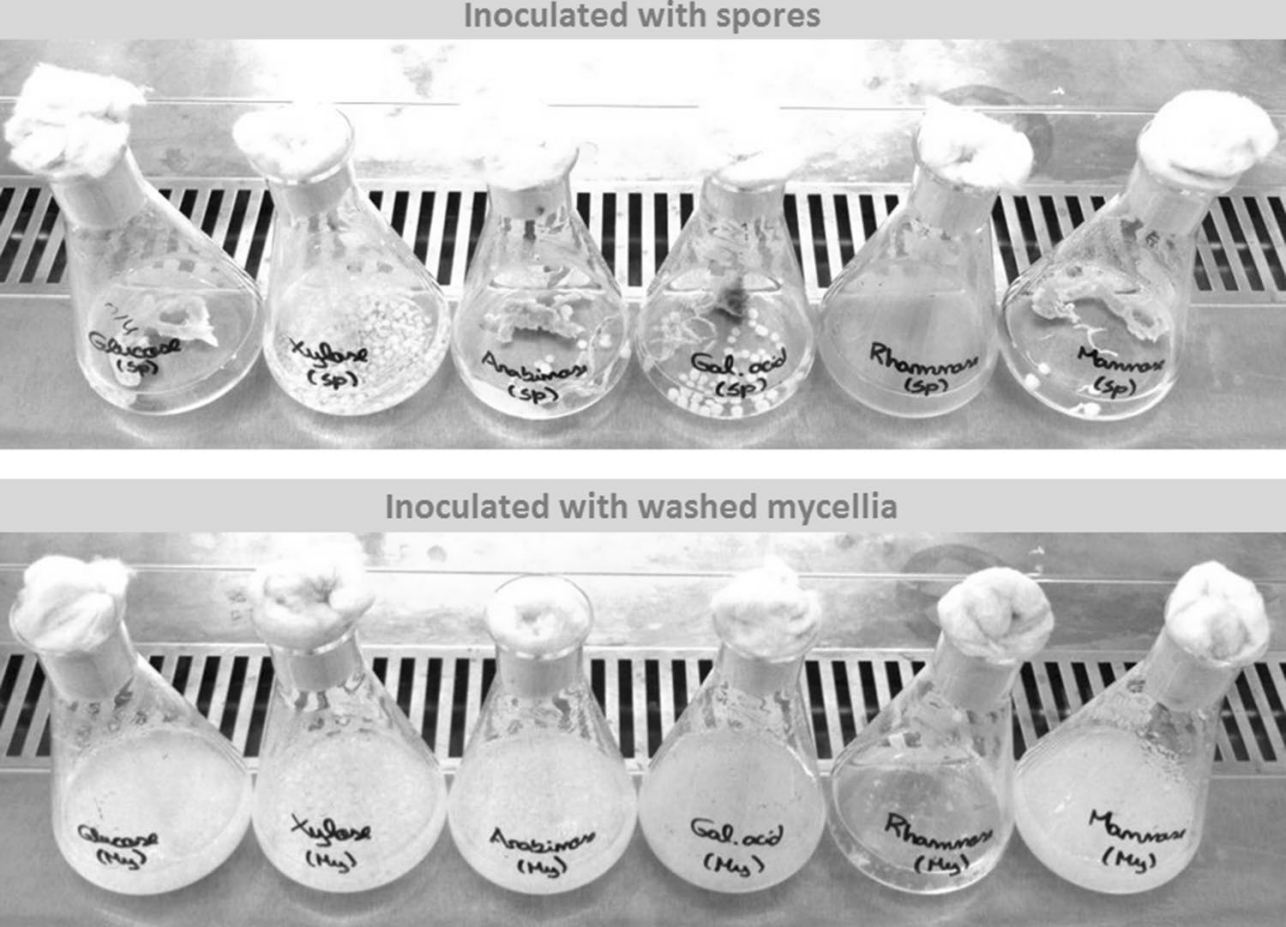
*A. niger* morphology after 50 h of growth in minimal media inoculated with spores (upper panel) and washed mycelia (lower panel). From left to right: glucose, xylose, arabinose, galacturonic acid, rhamnose, and mannose

As a preliminary experiment to investigate which carbon sources would sustain growth and whether growth on different carbon sources would lead to different morphologies, *A. niger* was cultivated on minimal medium in shake flasks with six different substrates. Two sets of shake flasks cultivations were carried out. The first set was inoculated with spores, while the second set was inoculated with mycelia pre-grown on the same minimal medium with sorbitol as the carbon source (Fig. 1).

After 50 h of incubation, all shake flask cultures showed evident growth with the exception of rhamnose. l-Rhamnose did not seem to induce spore germination nor further growth of pre-grown mycelia. Nevertheless, growth has been observed on l-rhamnose as a sole carbon source for *A. niger* in minimal media [17, 18].

Further comparison between the sets of shake flask cultivations indicated that inoculation with spores generates heterogeneity in the morphology of the mycelia among the different carbon sources with sometimes the formation of pellets, while inoculation with mycelia induces a uniform and similar, pellet free, morphology for all the different carbon sources (Fig. 1). Such pellet free morphology is preferred in kinetic studies to avoid diffusion limitation.

### Bioreactor batch cultivations on single carbon sources

**Fig. 2:**
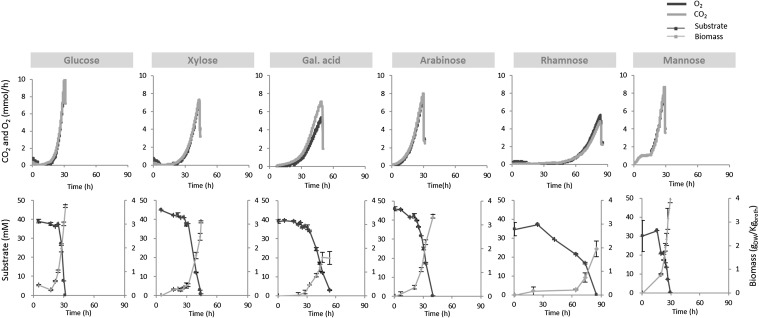
Carbon dioxide production and oxygen consumption rates (upper panel) and biomass production—right axis—and carbon-source consumption—left axis (lower panel) during batch phase using single different carbon sources

To be able to study growth and substrate uptake of *A. niger*, batch cultures were performed on each single substrate, in 2 L bench scale bioreactors. All bioreactor cultures were inoculated with washed mycelia pre-grown on minimal media with sorbitol as the carbon source. During the batch cultivations, samples were taken for quantification of the concentrations of biomass and residual carbon source. The concentrations of O_2_ and CO_2_ in the offgas were measured online for the calculation of the O_2_ consumption and CO_2_ production rates as a function of time. The obtained profiles for the different batch cultures are shown in Fig. 2.

In contrast to what was observed for the shake flask cultivations, *A. niger* did grow on minimal media with rhamnose as sole carbon source in the bioreactor.

The first striking differences between the batch cultivations carried out on the different carbon sources were the duration of the lag phase and the total duration of the batch. The rhamnose cultivation had the longest lag phase (around 50 h, and therefore explaining the absence of growth in shake flasks) and longest duration (85 h). In contrast, the glucose, arabinose and mannose cultivations had short or no lag phases and were finished after about 30 h.

It should be realized that the degree of reduction of the used carbon sources is not always the same [19]. For the sugars glucose, mannose, xylose, and arabinose, the generalized degree of reduction is four electrons per Cmol (*γ*_s_ = 4e^−^/C). However, galacturonic acid is more oxidized (*γ*_s_ = 3.333e^−^/C) and rhamnose is more reduced (*γ*_s_ = 4.333e^−^/C). This can clearly be seen from the respiratory quotients (Table 1), being the ratios of the biomass specific carbon dioxide production (*q*_C_^max^) and oxygen consumption (*q*_O_^max^) rates. As expected, for the substrates with a degree of reduction of 4e^−^/C, the respiratory quotients have a value slightly higher than one; however, for the more oxidized substrate galacturonic acid, the value is significantly higher than one, while for the more reduced substrate rhamnose, the value is significantly lower than one.

**Table 1:** Carbon and degree of reduction recoveries

Carbon source	RQ respiratory quotient	Carbon balance (%)	Degree of reduction balance (%)	*Y*_X/S_ Biomass yield (Cmol_X_/Cmol_S_ )	*μ* ^max^ max. growth rate (h^−1^)	*q*_S_^max^ max.substrate uptake rate (mol_S_ h^−1^)/Cmol_X)_	*q*_O_^max^ max.o_2_ uptake rate (${\text{mo}}{{\text{l}}_{{{\text{O}}_{\text{2}}}}}$h^−1^)/(Cmol_X_)	*q*_c_^max^ max.CO_2 production rate_ (${\text{mo}}{{\text{l}}_{{\text{C}}{{\text{O}}_{\text{2}}}}}$h^−1^)/Cmol_X)_
Glucose	1.03 ± 0.04	94 ± 2	94 ± 2	0.68 ± 0.03	0.221 ± 0.004	0.054 ± 0.001	0.101 ± 0.003	0.104 ± 0.003
Xylose	1.02 ± 0.07	87 ± 2	83 ± 2	0.77 ± 0.06	0.149 ± 0.007	0.039 ± 0.002	0.044 ± 0.002	0.045 ± 0.002
Arabinose	1.01 ± 0.04	98 ± 3	94 ± 5	0.47 ± 0.01	0.097 ± 0.001	0.041 ± 0.001	0.108 ± 0.003	0.109 ± 0.003
Gal acid	1.53 ± 0.07	93 ± 4	88 ± 10	0.50 ± 0.06	0.092 ± 0.005	0.031 ± 0.001	0.060 ± 0.002	0.092 ± 0.003
Mannose	1.00 ± 0.06	96 ± 2	92 ± 2	0.68 ± 0.03	0.151 ± 0.004	0.037 ± 0.001	0.072 ± 0.003	0.072 ± 0.003
Rhamnose	0.79 ± 0.05	101 ± 5	96 ± 7	0.67 ± 0.03	0.116 ± 0.003	0.029 ± 0.001	0.072 ± 0.003	0.057 ± 0.003

Reconciled biomass yield and maximum biomass specific conversion rates for different carbon sources in *A. niger* in single batch cultivation

The obtained measurements show in general that carbon and degree of reduction balances have recoveries of more than 90% (Table 1), indicating that by-product formation is either absent or insignificant.

The cumulative O_2_, CO_2_, substrate, and biomass data obtained during exponential growth (Online Resource 1) were used to obtain the best estimates of the biomass yield and biomass specific conversion rates (Table 1) using data reconciliation (explained in Materials and methods section). The biomass elemental composition was analysed for each substrate and taken into account for the data reconciliation (Online Resource 2).

Large differences in biomass yields and maximum biomass specific conversion rates were observed for the different single substrate cultivations. Glucose, xylose, and mannose were the substrates on which the highest biomass yield, maximum growth rate, and maximum substrate uptake rate were obtained.

From Table 1, the growth stoichiometry for unlimited exponential growth on the different substrates can be obtained by dividing the biomass specific conversion rates of substrate, oxygen, biomass (= maximum growth rate), and carbon dioxide by the maximum growth rate. This yields the overall reaction equations for the formation of 1 mol of biomass from substrate and oxygen:

$$\begin{aligned} & - 0.{\text{244 Glucose }} - 0.{\text{457 }}{{\text{O}}_{\text{2}}}+{\text{ 1 Biomas}}{{\text{s}}_{}}+{\text{ }}0.{\text{471 C}}{{\text{O}}_{\text{2}}}, \\ & - 0.{\text{262 Xylose }} - 0.{\text{295 }}{{\text{O}}_{\text{2}}}+{\text{ 1 Biomass }}+{\text{ }}0.{\text{3}}0{\text{2 C}}{{\text{O}}_{\text{2}}}, \\ & - 0.{\text{423 Arabinose }} - {\text{1}}.{\text{113 }}{{\text{O}}_{\text{2}}}+{\text{ 1 Biomass }}+{\text{ 1}}.{\text{124 C}}{{\text{O}}_{\text{2}}}, \\ & - 0.{\text{337 Galacturonic acid }} - 0.{\text{652 }}{{\text{O}}_{\text{2}}}+{\text{ 1 Biomass }}+{\text{ 1 C}}{{\text{O}}_{\text{2}}}, \\ & - 0.{\text{245 Mannose }} - 0.{\text{477 }}{{\text{O}}_{\text{2}}}+{\text{ 1 Biomass }}+{\text{ }}0.{\text{477 C}}{{\text{O}}_{\text{2}}}, \\ & - 0.{\text{25}}0{\text{ Rhamnose }} - 0.{\text{621 }}{{\text{O}}_{\text{2}}}+{\text{ 1 Biomass }}+{\text{ }}0.{\text{491 C}}{{\text{O}}_{\text{2}}}. \\ \end{aligned}$$

From an energy point of view, looking at the consumed oxygen per produced biomass, it is observed that xylose has the lowest oxygen consumption (about $0.30{\text{mol}}_{{\text{O}}_2}/{\text{mol}}_{\text{biomass}})$. Glucose and mannose have a higher oxygen requirement (about $0.47\;~{\text{mo}}{{\text{l}}_{{{\text{O}}_{\text{2}}}}}{\text{/mo}}{{\text{l}}_{{\text{biomass}}}}$), while galacturonic acid and rhamnose have an even higher oxygen demand of respectively 0.65 and $0.62\;~{\text{mo}}{{\text{l}}_{{{\text{O}}_{\text{2}}}}}{\text{/mo}}{{\text{l}}_{{\text{biomass}}}}$. Finally, arabinose has the highest oxygen consumption of $1.1\;~{\text{mo}}{{\text{l}}_{{{\text{O}}_{\text{2}}}}}{\text{/mo}}{{\text{l}}_{{\text{biomass}}}}$. These differences representing the differences in net energy expenditure for the formation of biomass for these different substrates may be due to different metabolic pathways. In case of arabinose, the energy and oxygen requirements are quite puzzling when compared to xylose, as this substrate has a very similar assimilation pathway in *A. niger*. These outputs ought to be further explored and confirmed by metabolic flux analysis.

### Bioreactor batch cultivations on a mixture of carbon sources

**Fig. 3:**
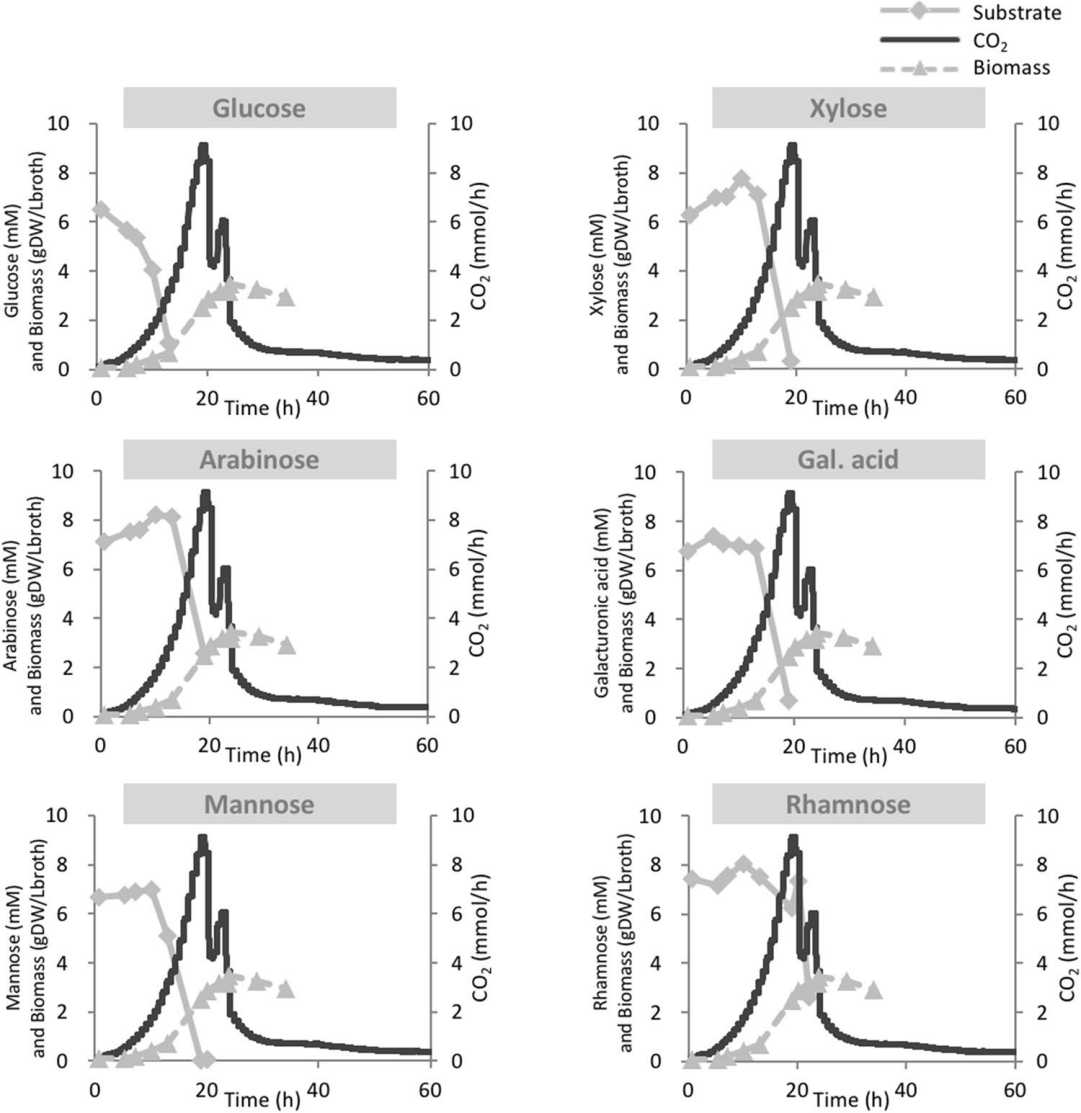
Carbon dioxide production rate and carbon source and biomass concentrations during batch phase in a mixture of six carbon sources

The metabolic versatility of *A. niger* was investigated during growth on a mixture of all six carbon sources. Remarkably, in this cultivation, the CO_2_ profile resembled the typical pattern of diauxic growth (Fig. 3). This could indicate that some of the substrates were taken up and metabolised simultaneously, while the uptake and metabolism of other substrates were repressed and occurred only after the repressing substrates were depleted.

Quantification of the residual concentrations of the different carbon sources at different timepoints during the batch cultivation was carried out with two methods (HPLC and GC-IDMS/MS), which gave essentially the same results. Here, we only show the results obtained with GC-IDMS/MS. We plotted each substrate in a different graph to facilitate visualization (Fig. 3). From these plots, it can be seen that glucose consumption started immediately, while the other carbon sources were not consumed during the first 10 h after inoculation, indicating that the presence of glucose repressed the metabolism of all other carbon sources. The repression of the transport and metabolism of less preferred substrates in the abundance of a sugar which is easily metabolized, e.g., glucose, is a well-known phenomenon amongst microorganisms, also in case of filamentous fungi [20–22].

After glucose was almost depleted, mannose consumption started. At the sixth sample point, when mannose was depleted, consumption of arabinose, galacturonic acid and xylose occurred. Finally, rhamnose consumption started only after 20 h of cultivation when all other carbon sources were depleted, which coincided with a sharp decrease of the O_2_ consumption and CO_2_ production rates. In the period during which rhamnose was consumed, the respiration rate increased again for a short period of time and decreased sharply after depletion of this last substrate.

During the batch cultivation, the specific growth rate can be obtained from the online measurement of the respiration rate, that is either oxygen consumption or carbon dioxide production. During exponential growth, the respiration rate also increases exponentially, and thus, from a plot of the natural logarithm of, e.g., the CO_2_ production rate, the growth rate can be obtained. From this, it was calculated that during the initial 10 h of the batch cultivation; when glucose was the only carbon source consumed, the specific growth rate was equal to 0.264 ± 0.005 h^−1^. Between 10 and 18 h, during the consumption of mannose, arabinose, galacturonic acid, and xylose, the growth rate was 0.201 ± 0.005 h^−1^ (Online Resource 3). The initial growth rate during the first 10 h on glucose was slightly higher than measured in the single substrate batch cultivation, while the growth rate obtained during consumption of mannose, arabinose, galacturonic acid, and xylose was significantly higher than the growth rates obtained from the single substrate batches (Table 1). This suggests that these carbon sources have individual uptake systems, which act in parallel.

### Sequential chemostat cultures

To obtain a more detailed insight in the kinetics of substrate uptake in *A. niger* at low carbon-source concentrations, a carbon-limited sequential chemostat experiment was performed on a mixture of the six substrates used for the single and mixed substrate batch cultivations. The experiment was carried out in a 7 L bioreactor designed for homogeneous chemostat cultivation of *A. niger*, without pellet formation and/or wall growth [8]. The feed medium of the chemostat was minimal medium complemented with an equimolar mixture of the six carbon sources, as described in the materials and methods.

After achieving steady state, the dilution rate was increased stepwise and the chemostat was run until a new steady state was obtained. The initial dilution rate was 0.04 h^−1^ which was sufficiently lower than the lowest maximum growth rate determined for the single substrate batch cultivations (Table 1), to enable growth on all carbon sources in carbon-limited chemostat cultures.

**Fig. 4:**
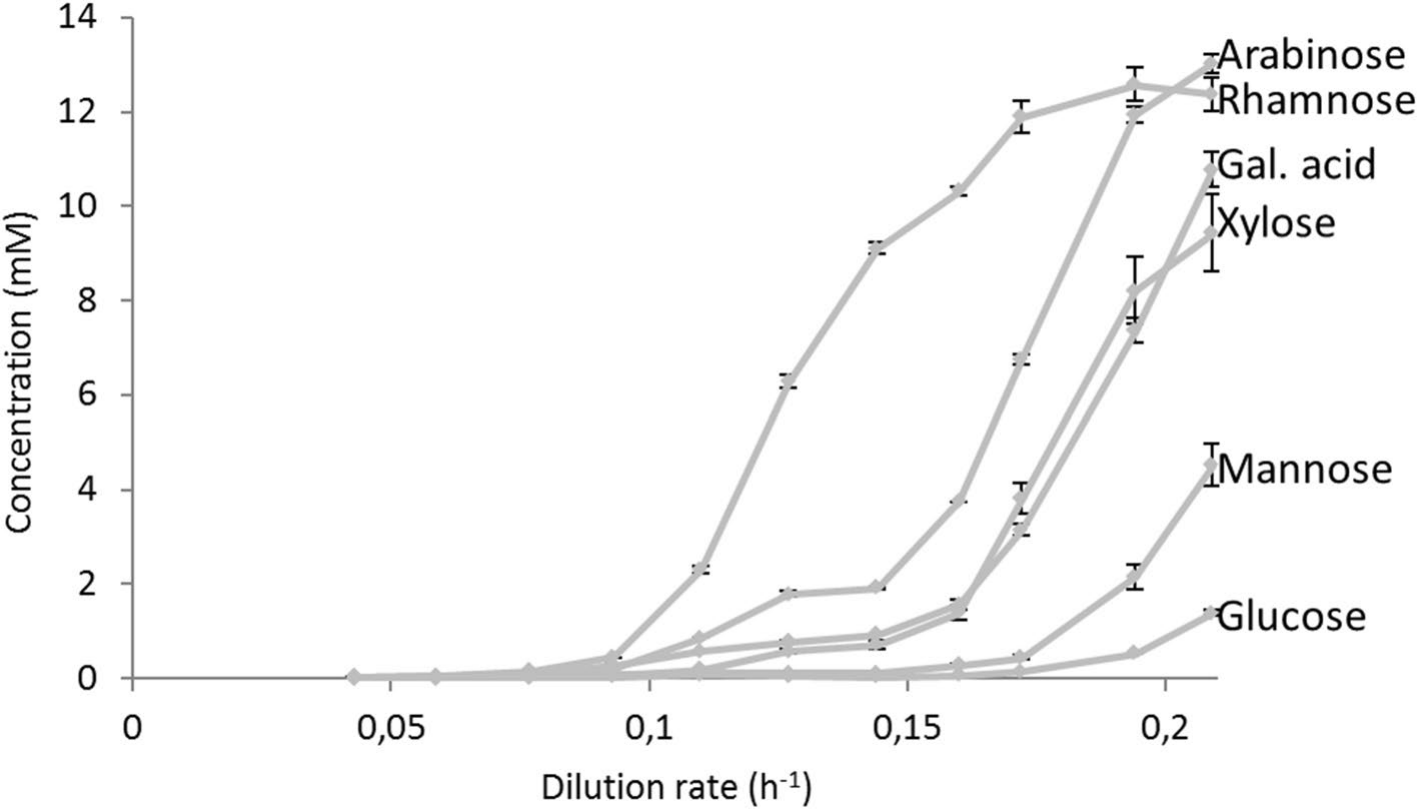
Residual concentration of carbon sources for different dilution rates during sequential chemostat cultures

**Fig. 5:**
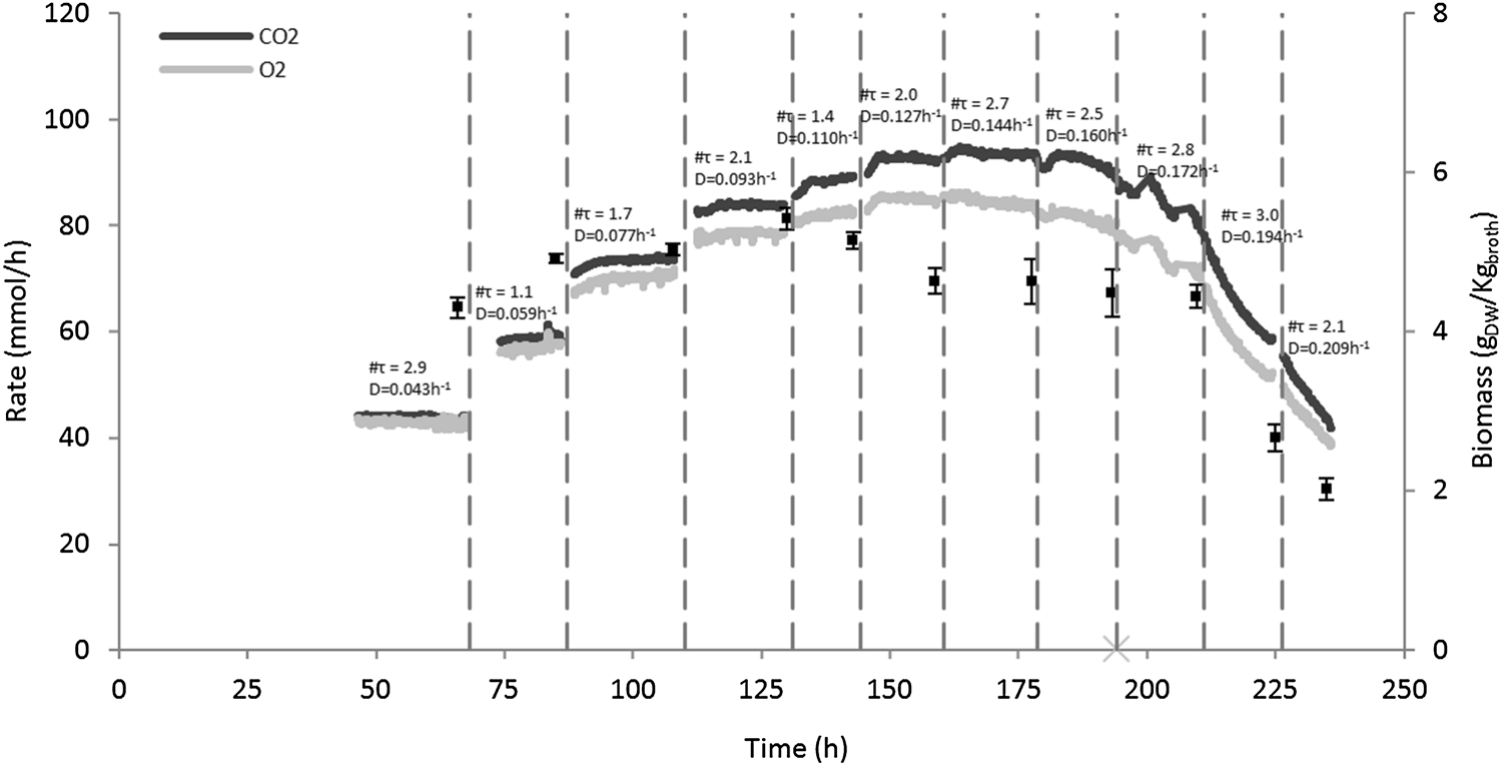
Oxygen and carbon dioxide consumption/production rates, biomass concentration (closed square), and number of residence times (#*τ*) during sequential chemostat cultivation

For all dilution rates, measurements of the concentrations of residual substrates (Fig. 4), biomass dry weight, and oxygen and carbon dioxide levels (Fig. 5) were performed. In addition, the total organic carbon (TOC) contents of the culture filtrate samples were determined. From these values, the measured amounts of residual substrates were subtracted to calculate the residual filtrate organic carbon, to verify whether by-products were formed (Online Resource 4). It was found that the in this way, determined residual carbon in the filtrate samples did not increase with increasing dilution rate, and was equal to an average value of 32 ± 8 mCmol/L, which was about 7% of the carbon supplied as mixed substrate to the culture. No attempts were undertaken to analyse the composition of the released carbon, as it was assumed that it mainly consisted of cell lysis products.

From the mentioned measurements (substrate, biomass, oxygen and carbon dioxide), it appeared that recoveries of carbon and degree of reduction were higher than 90% and thus the biomass specific rates were calculated and reconciled (Table 2, Online Resource 5).

**Table 2:** Biomass concentration, respiratory quotient, carbon and degree of reduction recoveries, and reconciled biomass specific net conversion rates at different dilution rates

Dilution rate (h^−1^)	0.04	0.06	0.08	0.09	0.11	0.13	0.14	0.16	0.17	0.19	0.21
Biomass concentration (*g*_DW_/*L*_broth_)	4.30 ± 0.13	4.92 ± 0.06	5.03 ± 0.07	5.42 ± 0.14	5.15 ± 0.11	4.63 ± 0.16	4.63 ± 0.29	4.48 ± 0.30	4.44 ± 0.14	2.66 ± .017	2.01 ± 0.14
RQ	1.03 ± 0.04	1.04 ± 0.04	1.07 ± 0.04	1.09 ± 0.04	1.10 ± 0.04	1.11 ± 0.04	1.14 ± 0.04	1.18 ± 0.04	1.19 ± 0.04	1.20 ± 0.05	1.16 ± 0.04
Carbon balance (%)	92 ± 2	95 ± 1	94 ± 1	91 ± 2	92 ± 1	89 ± 2	92 ± 2	93 ± 3	98 ± 1	97 ± 2	99 ± 1
Degree of reduction recovery (%)	86 ± 3	93 ± 2	88 ± 2	83 ± 3	87 ± 2	83 ± 2	87 ± 5	90 ± 3	97 ± 2	98 ± 2	102 ± 2
*q*_Glucose_ (mmol h^−1^)/Cmol_X_	3.05 ± 0.07	4.00 ± 0.06	5.14 ± 0.09	5.22 ± 0.09	6.62 ± 0.11	7.08 ± 0.09	8.78 ± 0.17	10.32 ± 0.21	12.66 ± 0.30	22.81 ± 1.12	31.39 ± 1.92
*q*_Xylose_ (mmol h^−1^)/Cmol_X_	3.67 ± 0.08	4.81 ± 0.07	6.19 ± 0.11	6.27 ± 0.11	7.91 ± 0.13	8.23 ± 0.11	10.09 ± 0.20	11.32 ± 0.25	11.41 ± 0.39	12.53 ± 1.26	15.72 ± 2.17
*q*_Arabinose_ (mmol h^−1^)/Cmol_X_	3.67 ± 0.08	4.80 ± 0.07	6.16 ± 0.11	6.21 ± 0.11	7.56 ± 0.13	7.54 ± 0.10	9.25 ± 0.18	9.38 ± 0.20	8.50 ± 0.25	5.98 ± 0.51	5.90 ± 0.76
*q*_Gal acid_ (mmol h^−1^)/Cmol_X_	2.80 ± 0.06	3.67 ± 0.06	4.70 ± 0.08	4.71 ± 0.09	5.83 ± 0.10	6.43 ± 0.10	7.46 ± 0.15	8.25 ± 0.18	8.56 ± 0.24	7.90 ± 0.61	2.21 ± 1.07
*q*_Mannose_ (mmol h^−1^)/Cmol_X_	3.31 ± 0.07	4.34 ± 0.07	5.58 ± .010	5.65 ± 0.10	7.17 ± 0.12	7.66 ± 0.10	9.50 ± 0.18	11.05 ± 0.22	13.48 ± 0.32	21.74 ± 1.12	25.60 ± 1.85
*q*_Rhamnose_ (mmol h^−1^)/Cmol_X_	3.35 ± 0.07	4.38 ± 0.07	5.59 ± 0.10	5.57 ± 0.10	6.07 ± 0.11	3.75 ± 0.09	3.24 ± 0.13	2.83 ± 0.14	1.75 ± 0.35	2.11 ± 0.71	3.97 ± 1.06
${q_{{{\text{O}}_{\text{2}}}}}$ (mmol h^−1^)/Cmol_X_	57.8 ± 2.1	72.7 ± 1.7	93.9 ± 2.6	85.2 ± 2.8	97.1 ± 2.9	78.1 ± 1.1	93.3 ± 3.6	95.0 ± 3.8	99.3 ± 3.7	129.4 ± 7.5	134.5 ± 9.2
${q_{{\text{C}}{{\text{O}}_{\text{2}}}}}$ (mmol h^−1^)/Cmol_X_	60.3 ± 2.1	76.1 ± 1.7	98.3 ± 2.6	89.9 ± 2.8	103.4 ± 2.9	86.5 ± 1.2	103.8 ± 3.7	3.7 ± 3.9	112.6 ± 3.9	143.8 ± 8.0	144.1 ± 9.4
*q*_X_ (mmol h^−1^)/Cmol_X_	42.5 ± 0.0	59.1 ± 0.0	76.6 ± 0.0	93.5 ± 0.0	109.7 ± 0.0	127.4 ± 0.0	143.9 ± 0.0	159.7 ± 0.0	172.0 ± 0.0	193.8 ± 0.0	209.2 ± 0.0
*q*_TOC_ (mmol h^−1^)/Cmol_X_	8.9 ± 0.2	11.1 ± 0.2	12.9 ± 0.2	5.9 ± 0.1	18.3 ± 0.5	14.4 ± 0.8	22.9 ± 1.2	1.2 ± 1.9	33.7 ± 3.6	82.4 ± 12.0	133.8 ± 20.4

From the quantification of the residual concentrations of the carbon sources in the chemostat, it can be seen that during the first four steady states, up to a dilution rate of 0.093 h^−1^, all six carbon sources were consumed simultaneously (Fig. 4). From the fifth steady state (*D* = 0.11 h^−1^), the residual concentrations of rhamnose, arabinose, and galacturonic acid started to increase, from *D* = 0.127 h^−1^ also xylose, from *D* = 0.16 h^−1^ mannose, and finally at *D* = 0.194 h^−1^ also glucose started accumulating.

At increasing dilution rate of the sequential chemostat fermentation, the increase of the residual concentrations of the different carbon sources occurred in the reverse order as the consumption of substrates in the multisubstrate batch fermentation. In these fermentations, l-rhamnose was only consumed after all other substrates were depleted, and appeared as the first increasing substrate at increasing dilution rate in the sequential chemostat cultivation. The observed increases of residual carbon-source concentrations also imply that the total amount of residual carbon source released from the chemostat via the effluent gradually increased with increasing dilution rate, and thus, the amount of consumed carbon gradually decreased. The result of this was that after an initial increase, the biomass specific respiration rate and steady-state biomass concentration levelled off, and finally decreased with increasing dilution rate (Fig. 5).

The observed increases of the residual concentrations of the different carbon sources indicate that the supply rate becomes higher than the uptake rate by the cells. In case of rhamnose, arabinose, galacturonic acid, and xylose, this already happened at biomass specific uptake rates of only 20% of the maximum value determined in the single substrate batch cultivations (see Tables 1, 2). In principle, this could have been caused by a very low affinity of the import systems for these substrates; however, it is to be expected that under carbon-limited chemostat conditions, high-affinity importers are expressed. Other possible explanations are catabolite repression and/or competition of multiple substrates for the same import system(s).

As mentioned above, glucose repression is likely to have occurred in the multisubstrate batch cultivations, as only after glucose was almost depleted the consumption of the other carbon sources started. In the carbon-limited chemostat cultivation, however, the residual glucose concentration was very low until a dilution rate of about 0.17 h^−1^ (Fig. 4). Nevertheless, the residual glucose concentration increased with increasing dilution rate (= growth rate) from 0.0125 mM at the lowest growth rate of 0.043 h^−1^ to 1.4 mM at the highest growth rate of 0.21 h^−1^ (Online Resource 6). If glucose repression would have been responsible for the limited uptake of the other carbon sources, this would imply that already at very low glucose concentrations repression of the uptake of the other substrates occurs. In [23], it was reported that in glucose limited chemostat cultivations of *Aspergillus nidulans*, CreA-mediated carbon catabolite repression increased with increasing dilution rate and thus specific growth rate. However, it follows from chemostat theory that the residual concentration of the growth limiting substrate increases with increasing dilution rate [24]. It can, therefore, not be excluded that the observed CreA-mediated carbon catabolite repression at higher dilution rates observed by [23]. was in fact caused by the onset of glucose repression due to the increase of the residual glucose concentration.

As the last substrate which was depleted, before rhamnose consumption started in the multi substrate batch was arabinose (Fig. 3), and the second substrate appearing with rhamnose was also arabinose (Fig. 4), arabinose is a possible candidate repressor of l-rhamnose transport/metabolism. It has indeed been observed that in *Aspergillus nidulans*, the α-l-rhamnosidase genes are strongly repressed by arabinose but also by glucose, ethanol, and sorbitol through a CreA-independent mechanism [18, 25]. It might be that the uptake and/or metabolism of the other substrates (arabinose, galacturonic acid, xylose and mannose) is repressed by glucose only, via a CreA-dependent mechanism. However, from the results of the multi substrate batch cultivations it appears that also mannose represses the metabolism of xylose, arabinose, and galacturonic acid. A better zoom-in on the kinetics and putative catabolite repression systems is advanced in the following section.

### Substrate uptake kinetics

**Fig. 6:**
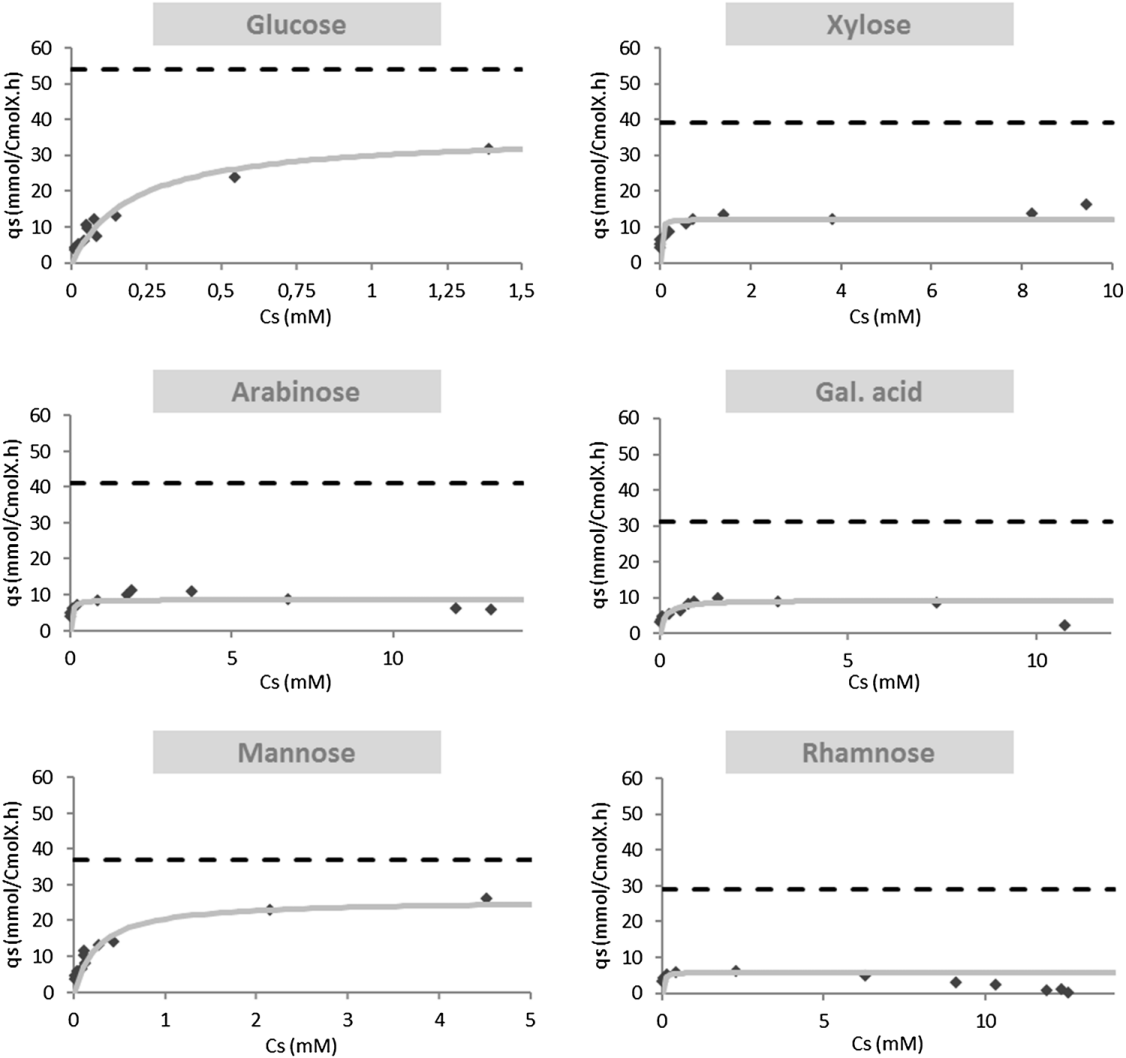
Biomass specific uptake rate of different substrates against residual substrate concentration during sequential chemostat cultivation. Maximum substrate uptake specific rate fitting using the Michaelis–Menten equation

To obtain a clearer picture on possible catabolite repression in *A. niger*, the biomass specific uptake rates of the different substrates in the sequential chemostat cultivations are plotted as a function of the residual substrate concentration (Fig. 6). In these plots, the maximum specific substrate uptake rate measured in the single substrate batch cultivations is indicated with a broken line. A Michaelis–Menten function (Eq. 3) was fitted to the data to obtain apparent *V*_max_ and *K*_m_ values for uptake of the different carbon sources in the multicarbon-source chemostat cultivation.

It can be seen from these plots that for all carbon sources, the maximum uptake rate is lower than observed in the single carbon-source batch cultivations. Especially, the uptake/metabolism of galacturonic acid, arabinose, and rhamnose seems to be repressed, as at a certain point the uptake rates of these carbon sources level off far below their *q*_S_^max^ values and subsequently decrease at increasing substrate concentration. In case of glucose, mannose, and xylose, repression does not seem to take place; however, with increasing substrate concentration, the specific substrate uptake does not reach the determined *q*_S_^max^. A possibility could be that glucose, mannose, and xylose are taken up by the same transport systems, resulting in competition between those substrates for the same transporters. Until now, three glucose import systems of *A. niger* have been characterized, namely, MSTA, MSTG, and MSTH. MSTA (*K*_m,glucose_ 0.025 ± 0.010 mM) also imports mannose (*K*_m_ 0.06 ± 0.02 mM) and xylose (*K*_m_ 0.3 ± 0.1 mM) [26]. MSTG (*K*_m,glucose_ 0.50 ± 0.04 mM), also imports mannose and possibly xylose, while MSTH (*K*_m,glucose_ 0.06 ± 0.005 mM) also imports mannose and fructose [27]. In addition, also three xylose import systems have been identified in *A. niger*, namely, XTLA, XTLB, and XTLC [18]. XTLA (*K*_m_ 0.09 ± 0.03 mM) also imports glucose (*K*_m_ 0.07 ± 0.01 mM) with the same *V*_max_ as for xylose. XTLB seems specific for xylose (*K*_m_ 15.0 ± 4.50 mM) and XTLC (*K*_m_ 4.71 ± 1.04 mM) imports glucose with a much higher affinity (*K*_m_ 0.11 ± 0.02 mM) and a roughly ten times higher *V*_max_ and should therefore be called a glucose importer.

From the published kinetic information on the six until now characterized sugar porters of *A. niger*, it appears that five of them are not very specific and import several sugars. In addition, in other filamentous fungi and yeasts, transport systems have been characterized that import several monosaccharides. In *Aspergillus nidulans*, the xylose transporter xtrD also imports glucose, galactose, and mannose [28], while the glucose transporters HxtB, HxtC, and HxtE also import fructose, mannose, and galactose [29]. A functional survey carried out by [30] found that the hexose transporter Hxt7 and galactose transporter GAL2 of *Saccharomyces cerevisiae* are able to import glucose, xylose, galactose, fructose, and mannose, while GAL2 in addition also imports ribose. Similar results were obtained for the sugar importers GXF1 and GXS1 of Candida intermedia, DEHA0D02167 and XylHP of *Debaryomyces hansenii*, and XUTt1 and XUT3 of *Scheffersomyces stipites*.

To model the uptake of glucose, mannose, and xylose, we assumed that these sugars are taken up by the same import systems and that their uptake could be described by competition (Eq. 4).

Both the specific uptake rates of glucose and mannose could be described well with this equation. For the specific uptake of xylose, no good fit could be obtained, indicating that besides competition probably also catabolite repression of xylose uptake and/or metabolism occurred (Online Resource 7).

The switch function (Eq. 5) was used to model glucose repression of the uptake of xylose, arabinose, galacturonic acid, and rhamnose in the sequential chemostat cultivation. The specific uptake rates of xylose, arabinose, and galacturonic acid could be described reasonably well by glucose repression (Online Resource 8); however, no fit could be obtained for the uptake of rhamnose (result not shown). As it is known that in *A. nidulans*, the α-l-rhamnosidase genes are strongly repressed by arabinose [23], and the same switch function (Eq. 5) was used to describe the repression of rhamnose uptake/metabolism by arabinose, which gave a reasonable fit (Online Resource 8). Further experiments using simultaneous substrate cultivation, i.e., with and without competitor/repressing substrates, would be advantageous to validate the mentioned hypothesis.

## Conclusions

Large differences in *q*_S_^max^ and *μ* ^max^ for individual substrates was observed, but in combination, and some carbon sources were consumed simultaneously and some sequentially. We found that the uptake of glucose, xylose, and mannose seems to be competing for the same transport systems, while the uptake of arabinose, galacturonic acid, and rhamnose appeared to be repressed by the presence of other substrates.

The stoichiometric and kinetic characterization of growth on different carbon sources, and the role of substrates as repressors or competitors during cultivation, should be considered in the design of a fermentation process based on plant waste feedstocks. In addition, the transition from simple carbon sources to more complex substrate mixtures requires careful attention.

## Electronic supplementary material

Below is the link to the electronic supplementary material.

Supplementary material 1 (DOCX 1171 KB)
