# Supplementary material for: Stoichiometry and kinetics of single and mixed substrate uptake in Aspergillus niger
Source: Bioprocess Biosyst Eng. 2017 Oct 19;41(2):157–70. doi: 10.1007/s00449-017-1854-3 (PMC5773628; doi:10.1007/s00449-017-1854-3)
Supplement: Supplementary file 1 — Supplementary material 1 (DOCX 1171 KB) [file 449_2017_1854_MOESM1_ESM.docx]

**Online Resource 1** Biomass and CO_2_ formation and oxygen and substrate consumption profiles for maximum biomass specific conversion rates estimation during exponential batch phase in different carbon sources. Unreconciled cumulative amounts (^▄^), reconciled rates (^▄^).

Online Resource 2 Biomass elemental composition, molecular weight and degree of reduction for growth in six different carbon sources.

|  | Elemental composition | Cmol weight (g/Cmol) | Degree of reduction |
| --- | --- | --- | --- |
| Glucose | C_1_ H_1.79_ N_0.14_ O_0.66_ P_0.015_ S_0.004_ | 27.93 ± 0.06 | 4.149 |
| Xylose | C_1_ H_1.77_ N_0.13_ O_0.68_ P_0.014_ S_0.003_ | 27.80 ± 0.19 | 4.108 |
| Arabinose | C_1_ H_1.75_ N_0.14_ O_0.66_ P_0.011_ S_0.003_ | 27.80 ± 0.06 | 4.083 |
| Galacturonic acid | C_1_ H_1.72_ N_0.14_ O_0.63_ P_0.011_ S_0.003_ | 27.17 ± 0.12 | 4.113 |
| Mannose | C_1_ H_1.75_ N_0.15_ O_0.65_ P_0.018_ S_0.004_ | 28.00 ± 0.00 | 4.114 |
| Rhamnose | C_1_ H_1.67_ N_0.12_ O_0.67_ P_0.009_ S_0.003_ | 27.64 ± 0.10 | 4.033 |

Online Resource 3 Carbon dioxide production rate and oxygen consumption rate during batch phase in a mixture of six carbon sources (left) and maximum growth rate determination (right): D= 0.201 ± 0.005 h^-1^.

Online Resource 4 Carbon concentration of residual carbon sources and calculated residual filtrate TOC for different dilution rates during sequential chemostat cultures.


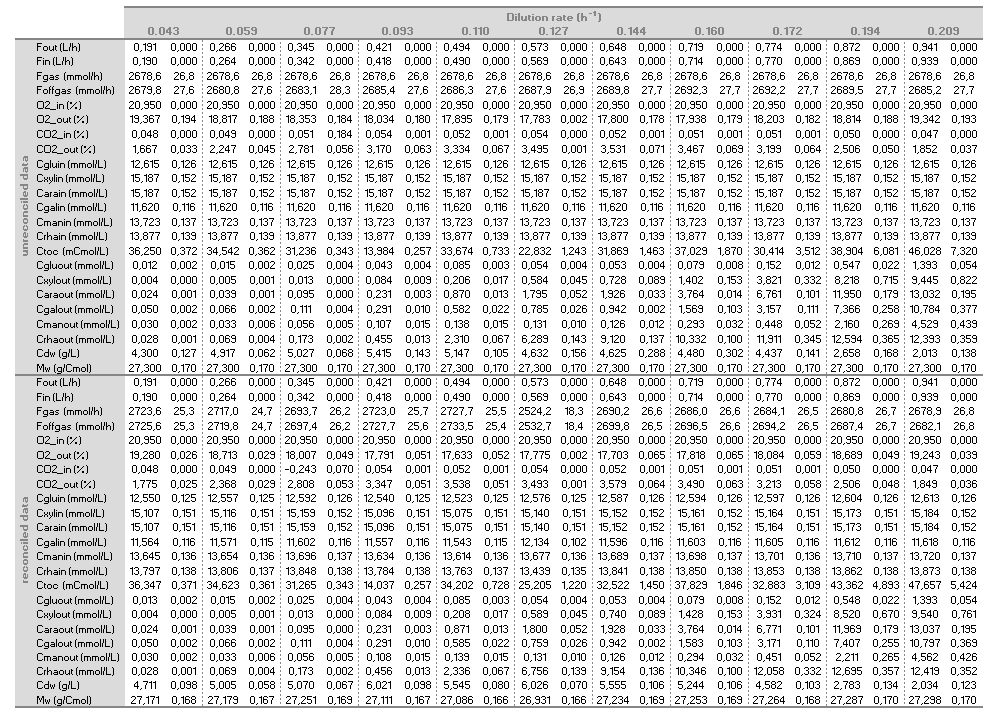
 Online Resource 5 Unreconciled and reconciled primary data at different dilution rates in the sequential chemostat cultures.

Online Resource 6 Residual substrate concentration for six carbon sources at different dilution rates in the sequential chemostat cultivation.

| Dilution rate (h^-1^) | Glucose (mM) | | | Xylose (mM) | | | Arabinose (mM) | | | Gal acid (mM) | | | Mannose (mM) | | | Rhamnose (mM) | | |
| --- | --- | --- | --- | --- | --- | --- | --- | --- | --- | --- | --- | --- | --- | --- | --- | --- | --- | --- |
| 0.043 | 0.012 | ± | 0.002 | 0.004 | ± | 0.000 | 0.024 | ± | 0.001 | 0.050 | ± | 0.002 | 0.030 | ± | 0.002 | 0.028 | ± | 0.001 |
| 0.059 | 0.015 | ± | 0.002 | 0.005 | ± | 0.001 | 0.039 | ± | 0.001 | 0.066 | ± | 0.002 | 0.033 | ± | 0.006 | 0.069 | ± | 0.004 |
| 0.077 | 0.025 | ± | 0.004 | 0.013 | ± | 0.000 | 0.095 | ± | 0.000 | 0.111 | ± | 0.004 | 0.056 | ± | 0.005 | 0.173 | ± | 0.002 |
| 0.093 | 0.043 | ± | 0.004 | 0.084 | ± | 0.009 | 0.231 | ± | 0.003 | 0.291 | ± | 0.010 | 0.107 | ± | 0.015 | 0.455 | ± | 0.013 |
| 0.110 | 0.085 | ± | 0.003 | 0.206 | ± | 0.017 | 0.870 | ± | 0.013 | 0.582 | ± | 0.022 | 0.138 | ± | 0.015 | 2.310 | ± | 0.067 |
| 0.127 | 0.054 | ± | 0.004 | 0.584 | ± | 0.045 | 1.795 | ± | 0.052 | 0.785 | ± | 0.026 | 0.131 | ± | 0.010 | 6.289 | ± | 0.143 |
| 0.144 | 0.053 | ± | 0.004 | 0.728 | ± | 0.089 | 1.926 | ± | 0.033 | 0.942 | ± | 0.002 | 0.126 | ± | 0.012 | 9.120 | ± | 0.137 |
| 0.160 | 0.079 | ± | 0.008 | 1.402 | ± | 0.153 | 3.764 | ± | 0.014 | 1.569 | ± | 0.103 | 0.293 | ± | 0.032 | 10.332 | ± | 0.100 |
| 0.172 | 0.152 | ± | 0.012 | 3.821 | ± | 0.332 | 6.761 | ± | 0.101 | 3.157 | ± | 0.111 | 0.448 | ± | 0.052 | 11.911 | ± | 0.345 |
| 0.194 | 0.547 | ± | 0.022 | 8.218 | ± | 0.715 | 11.950 | ± | 0.179 | 7.366 | ± | 0.258 | 2.160 | ± | 0.269 | 12.594 | ± | 0.365 |
| 0.209 | 1.393 | ± | 0.054 | 9.445 | ± | 0.822 | 13.032 | ± | 0.195 | 10.784 | ± | 0.377 | 4.529 | ± | 0.439 | 12.393 | ± | 0.359 |


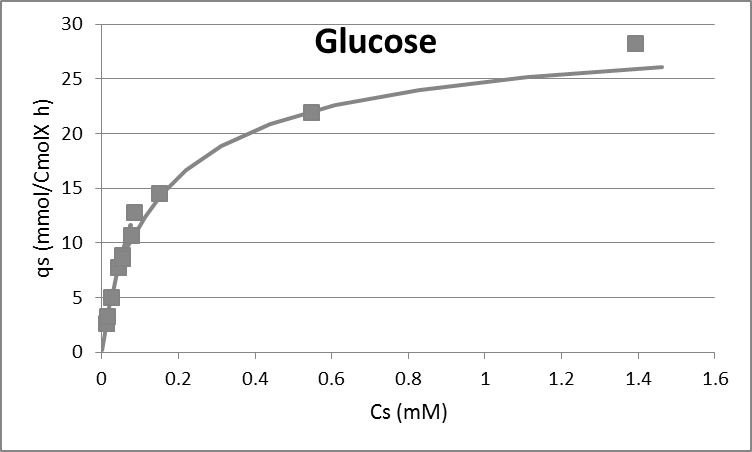


A


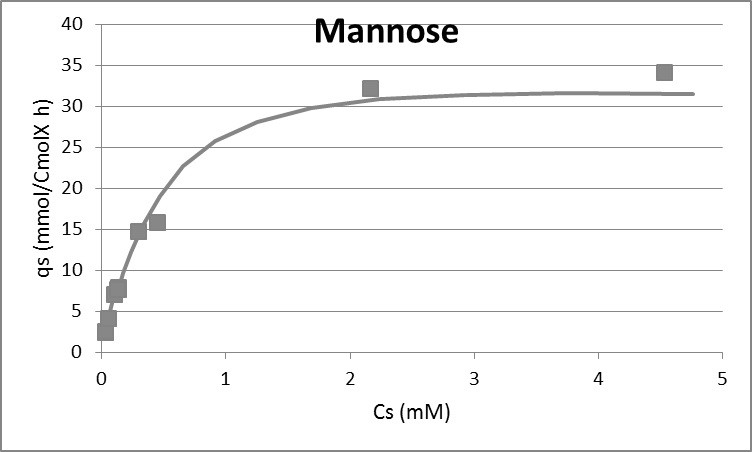


B


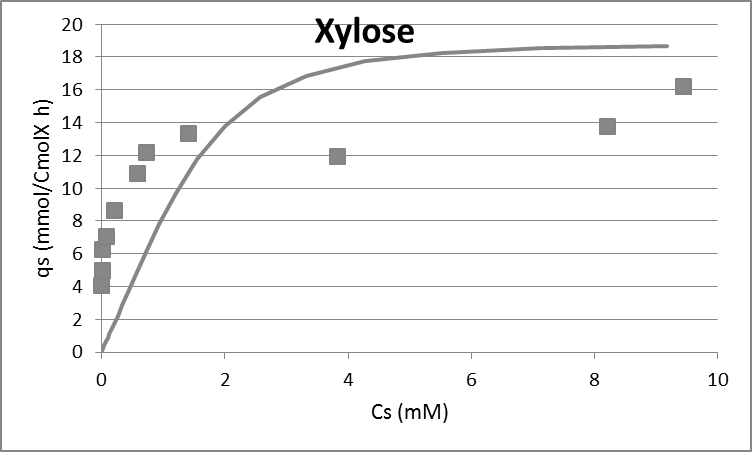


C

Online Resource 7 The biomass specific uptake rates of glucose (A), mannose (B) and xylose (C) in the multi substrate sequential chemostat cultivation, plotted against their respective residual concentrations. The solid lines represents the modelling of the uptake rates of the three substrates according to competition for the same transport system.

*
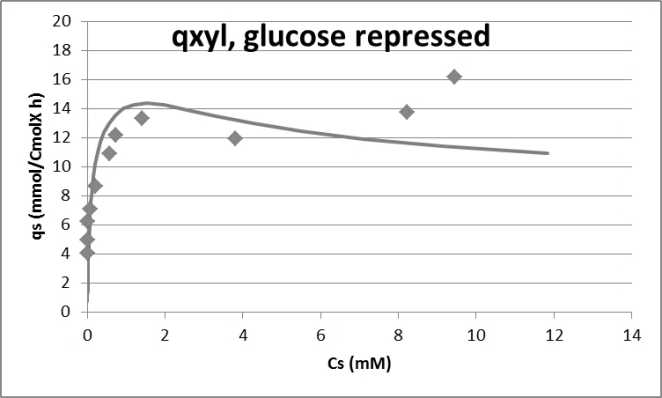
*

A

*
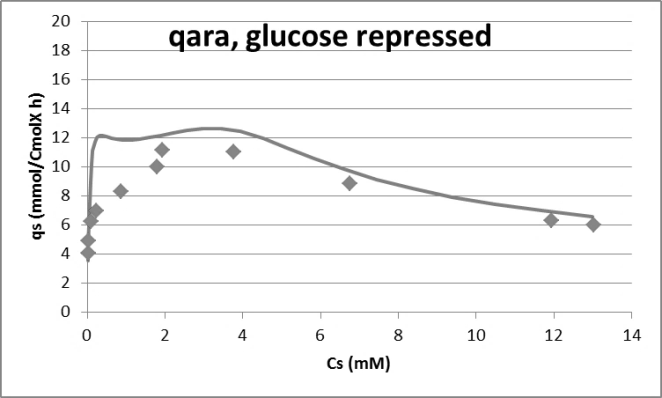
*

B

*
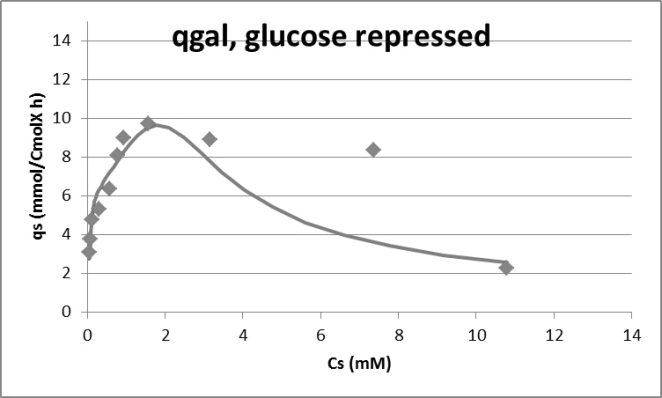
*

C


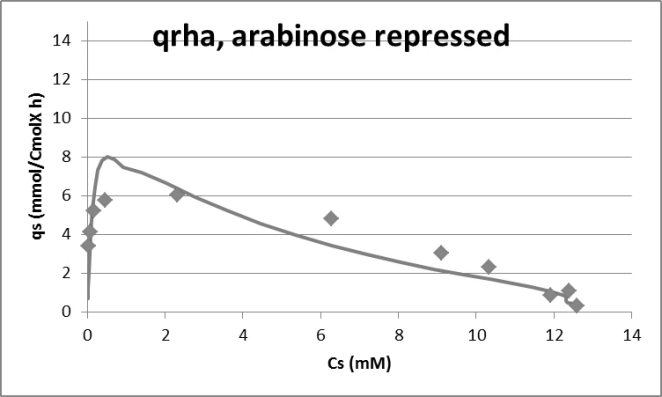


D

Online Resource 8 The biomass specific uptake rates of xylose (A), arabinose (B) galacturonic acid (C) and rhamnose (D) in the multi substrate sequential chemostat cultivation, plotted against their respective residual concentrations. The solid lines represents the modelling of the uptake rates according to catabolite repression by glucose (in case of xylose, arabinose and galacturonic acid) and arabinose (in case of rhamnose).
